# Randomised control trial on the sustained carry-over effects of pulsed electromagnetic field therapy for the treatment of Achilles tendinopathy

**DOI:** 10.1038/s41598-026-38596-3

**Published:** 2026-02-06

**Authors:** Violet Man-Chi Ko, Sai-Chuen Fu, Patrick Shu-Hang Yung, Samuel Ka-Kin Ling

**Affiliations:** 1 Department of Physical Education and Sports Science, National Institute of Education, Nanyang Technological University, Singapore, Singapore; 2 Department of Orthopaedics and Traumatology, Faculty of Medicine, The Chinese University of Hong Kong (CUHK), Shatin, Hong Kong SAR China; 3 Center for Neuromusculoskeletal Restorative Medicine, The Chinese University of Hong Kong (CUHK), Shatin, Hong Kong SAR China

**Keywords:** PEMF, VISA-A, NPRS, Pain, Function, Rehabilitation, Rehabilitation, Tendons

## Abstract

Achilles tendinopathy is a common musculoskeletal condition characterised by pain and functional impairment. Current treatments, such as eccentric exercises, are recommended as first-line options, but their success rates are often disappointing. Adjunctive novel treatments may help improve outcomes for these patients. This study aims to determine whether participants receiving active pulsed electromagnetic field (PEMF) therapy combined with eccentric exercise experience sustained additional improvements compared to those receiving sham PEMF therapy combined with eccentric exercise. A total of 65 participants were recruited and randomly assigned to an 8-week programme of either active PEMF therapy or sham PEMF therapy. Additionally, all participants completed a standardised home-based eccentric exercise programme for 12 weeks. The Victorian Institute of Sport Assessment questionnaire was the primary outcome. The Numeric Pain Rating Scale and the Short Form 36 were selected as the secondary outcomes. An eight-week PEMF therapy, conducted alongside a twelve-week eccentric exercise regimen, resulted in notable improvements for both treatment groups across all outcomes. These improvements were maintained at the twenty-six-week follow-up. PEMF therapy constitutes an innovative, non-invasive therapeutic approach that shows potential for treating Achilles tendinopathy. Additional research into the optimal dosage of PEMF therapy is recommended to develop thorough future clinical trials.

Trial registration: ClinicalTrials.gov (NCT05316961). Registered on 7th April 2022.

Protocol Registration: The study protocol was published in Trials (10.1186/s13063-023-07434-6).

## Introduction

Achilles tendinopathy is a common orthopaedic condition that affects both athletes and sedentary populations, disrupting training and competition schedules for professional athletes, and reducing working hours, income, and quality of life for recreational athletes and middle-aged, overweight individuals^1^. The annual incidence of Achilles tendon overuse injuries can reach up to 9% among elite athletes, particularly those engaged in prolonged, high-intensity physical activities such as middle- and long-distance running, track and field, and various ball sports^2,3^. Among the non-athletic populations, the condition is reported more frequently in males than in females, potentially attributable to differences in physical activity level^4^.

The actual cause of Achilles tendinopathy remained unknown, and various risk factors have been suggested^5,6^. Common intrinsic factors are age, gender, and weight. Extrinsic factors can predispose athletes to Achilles tendinopathy, such as changes in the training regimen, improper technique, history of injuries, footwear, and poor training environment. The primary pathological cause of tendinopathy is excessive loading of tendons during intense physical activity, which results from an imbalance between muscle strength and tendon elasticity^7^. The repetitive super-physiologic overload on the Achilles tendon can lead to tendon sheath inflammation, tendon body degeneration, or a combination of both^7^. Pain and discomfort induced by exercise may become persistent and manifest even during routine daily activities^2^.

A range of conservative treatments exist, but there is no definitive gold standard for the treatment approach^8,9^. Eccentric exercise is recommended to be the initial treatment for patients with Achilles tendinopathy, but its therapeutic effects on pain reduction are not satisfactory^10,11^. Developing a novel treatment adjunct to eccentric exercise for managing Achilles tendinopathy is essential.

Pulsed electromagnetic field (PEMF) therapy could be a potential treatment adjunct for Achilles tendinopathy. PEMF therapy is conventionally used to manage pain associated with musculoskeletal disorders, such as knee osteoarthritis and low back pain, in clinical settings^12^. Besides its safety profile, PEMF therapy might also promote healing in tendon injuries. Denaro et al. investigate the effects of PEMFs on human tenocyte cultures and assess whether PEMFs represent a viable therapeutic option in tendon pathologies^13^. Compared with the control group, exposure to PEMF significantly accelerates cut closure 12 and 24 h after the injury. The study results provide the preliminary evidence to support the study of the effects of PEMFs on tendinopathies^13^.

Currently, there are no standardised guidelines regarding the frequency and intensity utilised in applications for musculoskeletal disorders^14^. Low-intensity PEMF treatment produces optimal results in healthy human tendon cell cultures by promoting cell proliferation, increasing the expression of tendon-specific genes, and stimulating the release of anti-inflammatory cytokines and growth factors^15^. Additionally, repeated application of this low-intensity treatment causes human tendon cells to significantly increase their IL-10 production, which is vital in anti-inflammatory pathways^15^.

Therefore, this randomised controlled trial investigates whether participants receiving active PEMF therapy will have higher VISA-A scores than those receiving sham PEMF therapy. The clinical effects of PEMF therapy combined with eccentric exercise are assessed by a physiotherapist at baseline, 4, 8, 12, and 26 weeks. This article reports the carry-over effects of PEMF combined with eccentric exercise, with the short-term outcomes of this trial published in a peer-reviewed scientific journal^16^.

## Methods

### Study design

This study was a double-blind, parallel, randomised, placebo-controlled trial to assess the clinical effects of PEMFs in treating Achilles tendinopathy. Eligible participants were randomised to the intervention group or the control group. The trial was conducted at the teaching hospital of the Chinese University of Hong Kong, Prince of Wales Hospital. Participants received 8-week PEMF therapy and engaged in home-based eccentric exercises during the first 12 weeks. Participants did not receive any treatment after 12 weeks and had no restrictions on daily activities throughout the study period. The full study protocol was published in a peer-reviewed scientific journal^17^.

### Recruitment

The Joint Chinese University of Hong Kong - New Territories East Cluster Clinical Research Ethics Committee (Reference number: 2021.150) approved clinical research ethics for this study. All methods were performed in accordance with the relevant guidelines and regulations. After gaining written informed consent from participants, the same musculoskeletal physiotherapist from CUHK conducted all assessments and treatment sessions at the Sports Injury and Biomechanics Laboratory from the Department of Orthopaedics and Traumatology at the Prince of Wales Hospital in Hong Kong. The orthopaedic surgeons and physiotherapist from the Department of Orthopaedics and Traumatology, Chinese University of Hong Kong (CUHK) conducted eligibility assessments according to the inclusion and exclusion criteria in Table 1.

**Table 1:** Inclusion and exclusion criteria.

Inclusion criteria
(1) Age between 18 and 70 (2) Focal clinical signs of Achilles tendinopathy with localised tenderness on palpation of the Achilles tendon (3) Achilles tendon pain or stiffness at rest and during exercise in the past three months (4) Increased tendon thickness or neovascularity was shown via ultrasound examination (5) Written informed consent
Exclusion criteria
(1) History of major injury or surgery on the affected lower limb in the past year (2) Mental or physical limitations hindering participant’s ability to complete assessments (3) With medical or musculoskeletal problems that could affect the ability to complete assessments and intervention (4) With active electronic implants like pacemakers and defibrillators (5) Fractures of the affected lower limb within the past 12 months

### Randomisation

The PEMF device supplier, Quantum TX, randomised the allocation into 1:1 blocks of 10 using an online randomiser (https://www.randomizer.org/).^18^ The company chief engineer allocated unique radio frequency identification (RFID) cards for physiotherapist from CUHK to arrange treatment sessions so that the assessors and participants could be blinded to the randomisation. The PEMF device was pre-programmed to recognise if it should deliver active or sham PEMFs based on the unique RFID card.

### Intervention

**Fig. 1:**
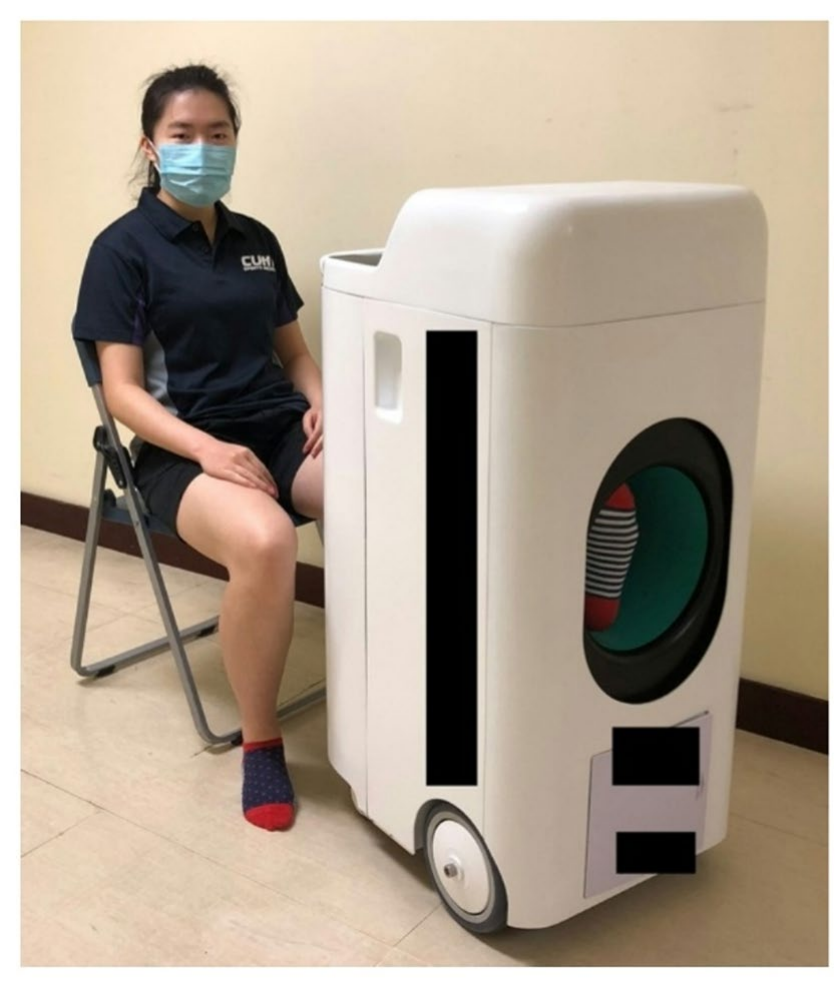
Sample photo of the setting during PEMF therapy. Informed consent was obtained to publish the information/image(s) in an online open-access publication. Participants are instructed to place their entire foot and ankle within the solenoid of the PEMF device.

**Fig. 2:**
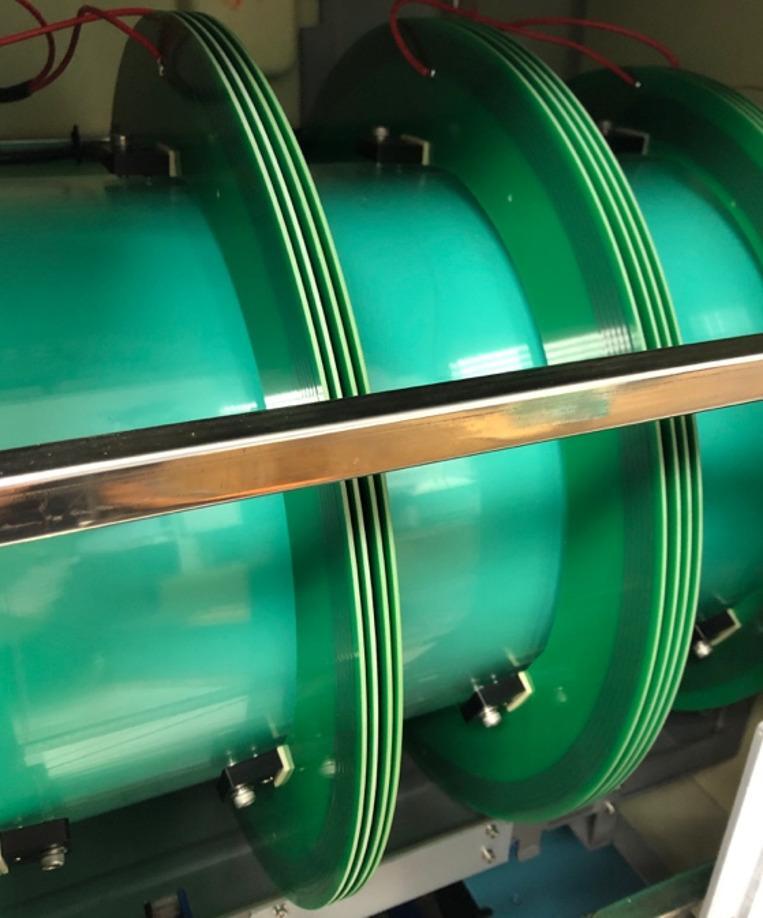
Interior design of the PEMF device. The solenoid design, featuring multiple coils, ensures an even distribution of PEMFs within the device.

All participants completed 16 PEMF sessions, with two sessions weekly over eight weeks. They were randomly assigned to either the experimental group, which received active PEMF therapy (1 mT, 50 Hz), or the control group, which received sham PEMF therapy (0 mT, 0 Hz). During each 10-minute session, all participants rested their entire foot and ankle within the solenoid of the PEMF device while seated (Fig. 1). The solenoid design, featuring multiple coils, ensures an even distribution of PEMFs within the PEMF device (Fig. 2). As long as the foot and ankle are placed within the solenoid, they are exposed to uniform PEMFs. In addition to the 8-week PEMF therapy, all participants performed daily home-based eccentric exercises for 12 weeks. Physiotherapists provided instructions on eccentric exercises after the first PEMF therapy session.

### Outcome measures

The primary outcome was the Victorian Institute of Sports Assessment – Achilles (VISA-A) questionnaire score, explicitly designed for Achilles tendinopathy^19^. Participants reported the level of pain, functional impairments and sporting activities. In order to reflect the actual clinical conditions of local patients in Hong Kong, the VISA-A was cross-culturally adapted before the start of this trial^19^. In addition, participants rated the level of pain using NPRS under two conditions: general on the day of review (NPRS general) and worst pain on the day of review (NPRS worst). Participants might not feel pain on the day of review, but they may experience tendon pain triggered by specific loading movements. The Short Form 36 health survey (SF36) was used to evaluate health-related quality of life^20^. All outcome measures were assessed at baseline, 4-week, 8-week, 12-week, and 26-week follow-up assessments.

### Sample size

According to a previous study using laser therapy combined with eccentric exercise for Achilles tendinopathy, we found that a minimum increase of 16 points in the VISA-A scores would confirm efficacy^21^. The power analysis to identify the minimal clinically important difference (MCID) was performed using G*Power software, with a 5% Type I error rate and 80% statistical power (correlation coefficient, 0.03; pooled standard deviation 20.25)^21^. The calculation indicated that 20 participants per group are required, adjusted for a 20% attrition rate, to detect a significant difference in VISA-A scores using a 2-way repeated-measures ANOVA. As a result, the final sample size was set at 40 participants.

### Statistical analysis

The data collected was analysed using SPSS, Version 29.0 (IBM). Repeated measures ANOVA was used to assess all data collected during baseline, week 4, week 8, week 12, and week 26 assessments. Participants should complete all assessment time points to be included in the data analysis. The significance level was set at *p* < .05.

**Fig. 3:**
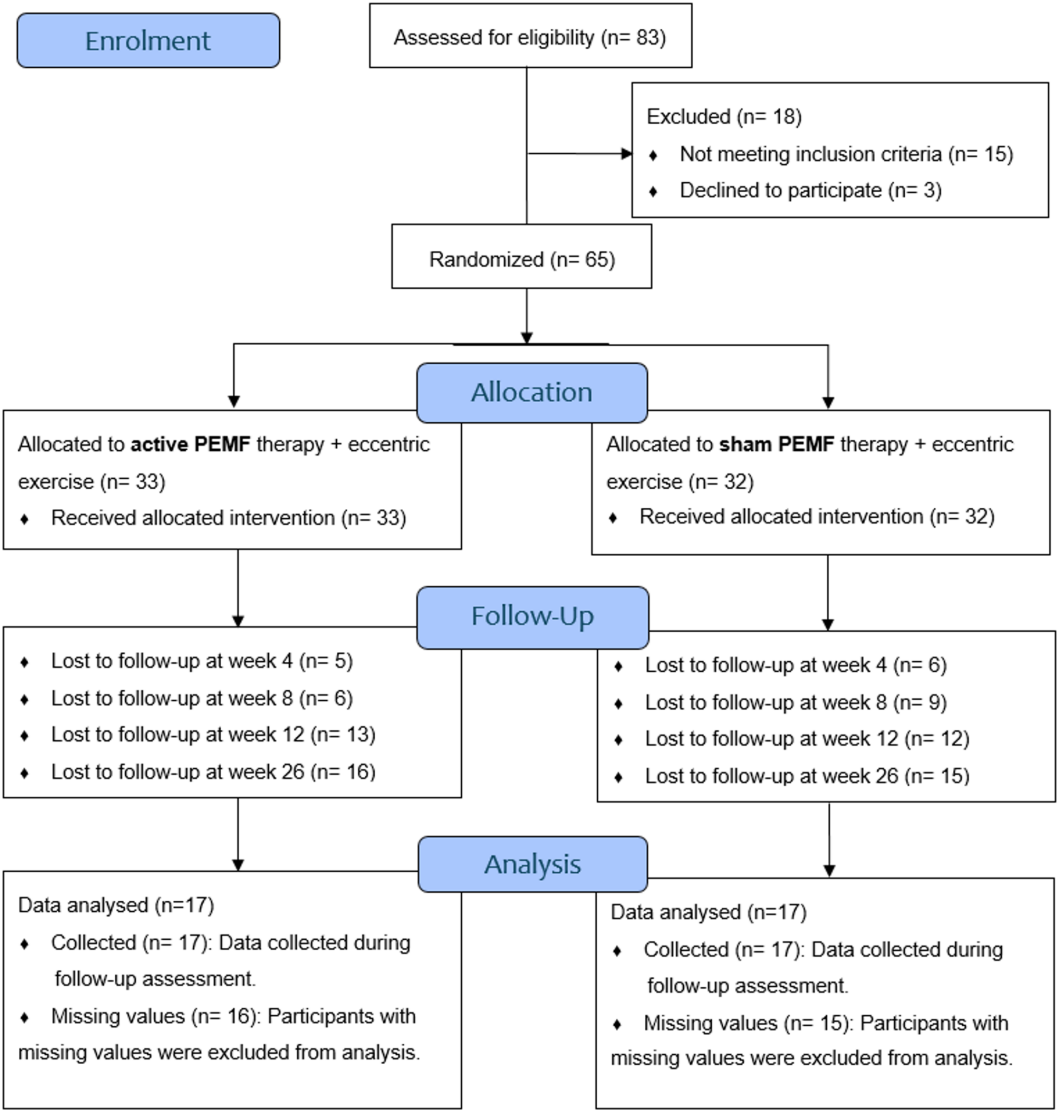
Flowchart of the study population.

## Results

Eighty-three participants were screened for eligibility between July 2021 and February 2023 (Fig. 3). After eligibility screening, 65 participants were recruited and randomly assigned to either the PEMF (*n* = 33) or sham (*n* = 32) groups. Of the eligible participants, 34 completed all follow-up assessments at weeks 4, 8, 12, and 26. These participants’ data were analysed using repeated measures ANOVA to identify significant differences between groups in VISA-A scores, Numeric Pain Rating Scale - general of the day of review (NPRS-general), Numeric Pain Rating Scale – worst pain on the day of review (NPRS-worst), and SF36. Tables 2 and 3 display the baseline characteristics and data analysis results across all outcome measures. No adverse effects were reported during the study.

**Table 2:** Baseline characteristics of participants.

Age, years	PEMF group (*n* = 17)	Sham Group (*n* = 17)
	54.12 ± 9.11	58.00 ± 10.09
Gender, female	5 (29.4%)	12 (70.6%)
Height, cm	164.15 ± 8.05	160.94 ± 12.82
Weigh, kg	68.95 ± 13.55	67.59 ± 20.90
BMI	25.54 ± 4.25	26.07 ± 7.33
Injured limb, right side, n (%)	9 (52.9%)	14 (82.4%)
Duration of symptoms, month	18.35 ± 17.45	38.47 ± 40.09
Type, midportion	4 (23.5%)	6 (35.3%)

^Data are reported as mean SD or n (%). BMI, body mass index; Type, type of tendinopathy.

### Victorian institute of sports assessment – achilles (VISA-A)

**Fig. 4:**
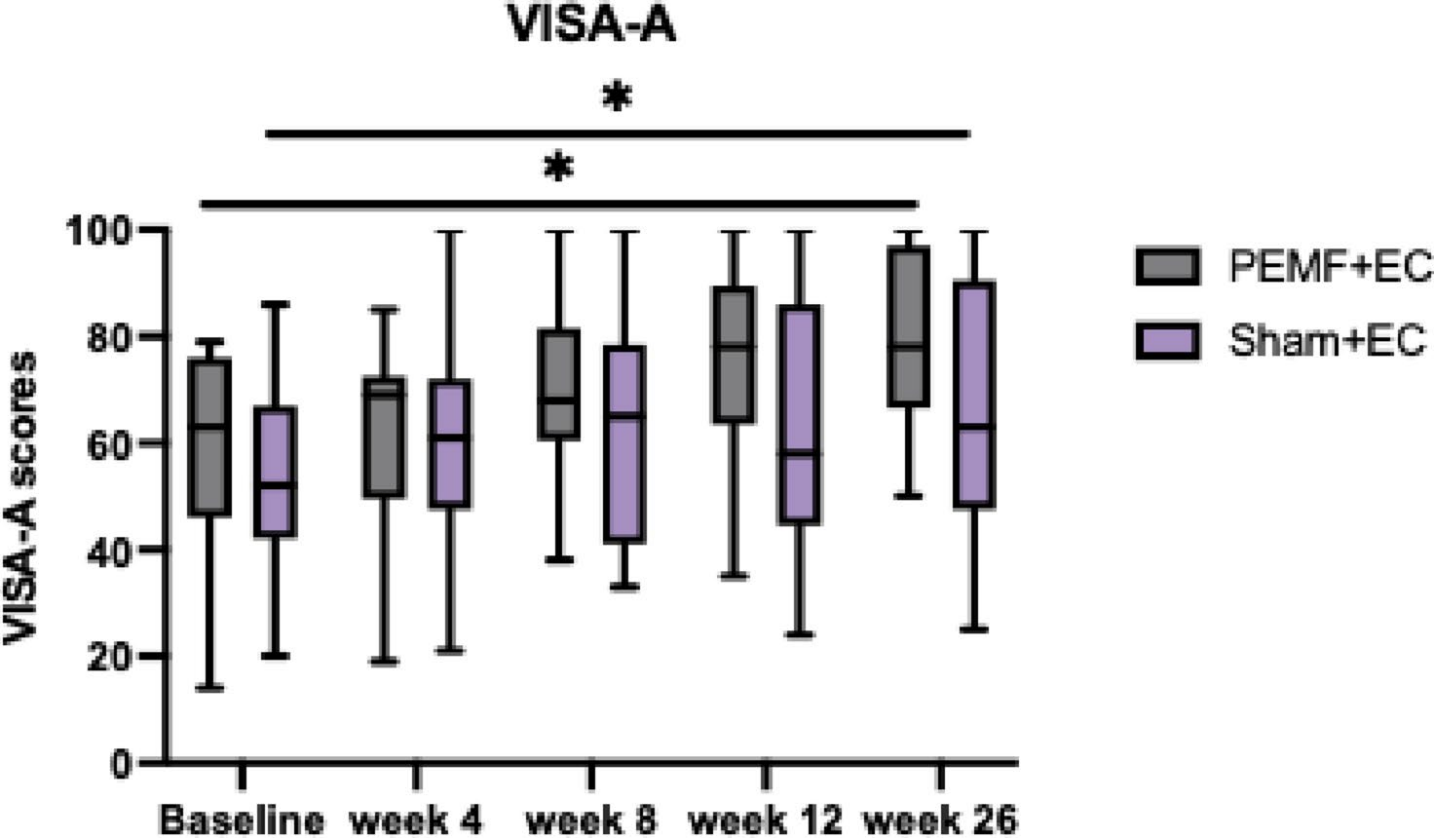
VISA-A. Comparison of VISA-A scores for participants across all assessment timepoints using repeated ANOVA. The data shown represent the median, interquartile range, PEMF group (*n* = 17), and Sham group (*n* = 17). VISA-A scores were significantly improved in both treatment groups after PEMF therapy. *Represents statistically significant difference within treatment groups.

There was no significant difference in the interaction effects (F = 1.427, *P* = .243), but there was a significant improvement within groups (F = 9.839, *P* < .001) (Fig. 4). Pre-treatment VISA-A scores significantly improved for treatment groups at week 26 (Table 3). The mean VISA-A scores for the PEMF group improved from 59.00 to 79.41 at week 26, whereas the Sham group improved from 53.65 to 64.41 during the same period.

### Numeric pain rating scale (NPRS)

**Fig. 5:**
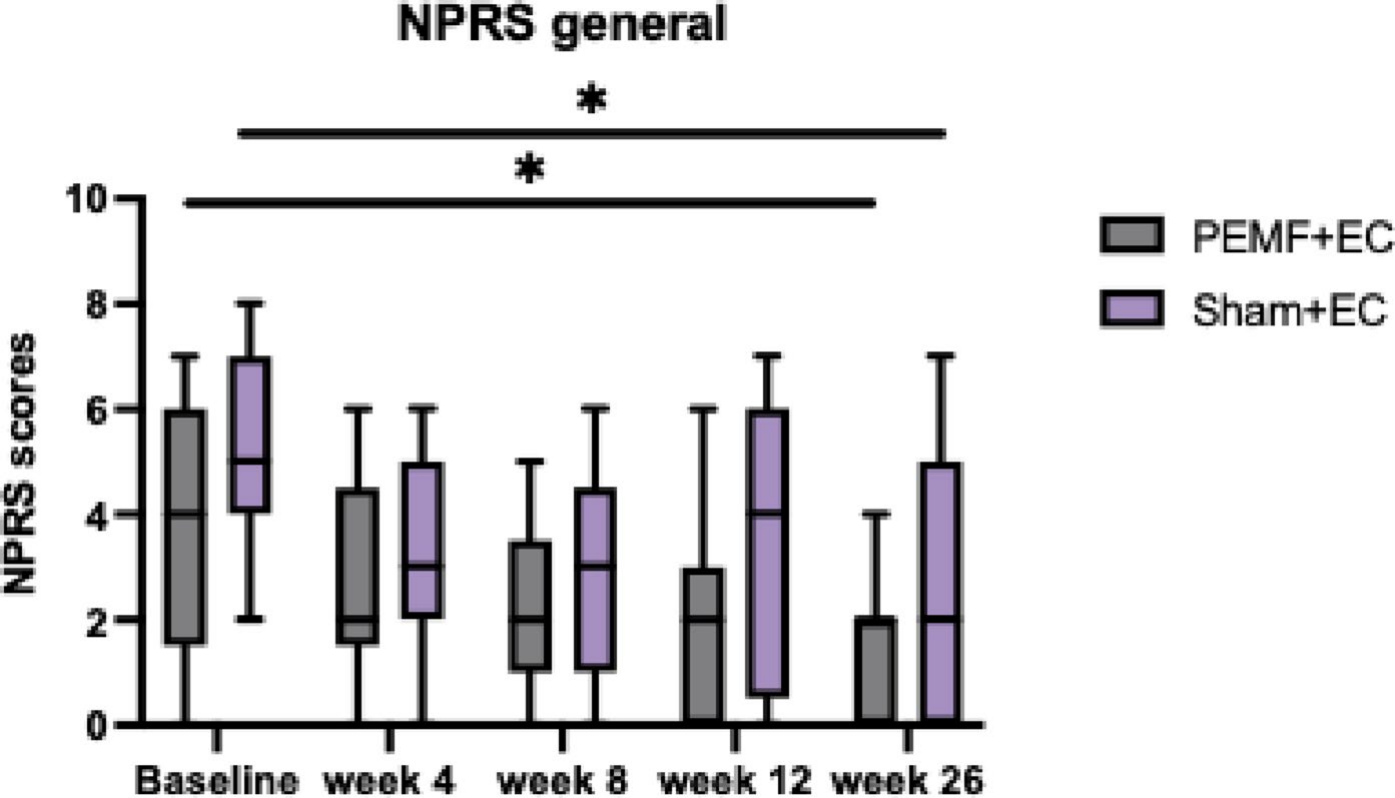
NPRS general. Comparison of NPRS general scores between PEMF and Sham groups across all assessment timepoints using repeated ANOVA. The data shown represent the median, interquartile range, PEMF group (*n* = 17), and Sham group (*n* = 17). NPRS general scores significantly decreased within both treatment groups after PEMF therapy. * Represents statistically significant difference within treatment groups.

**Fig. 6:**
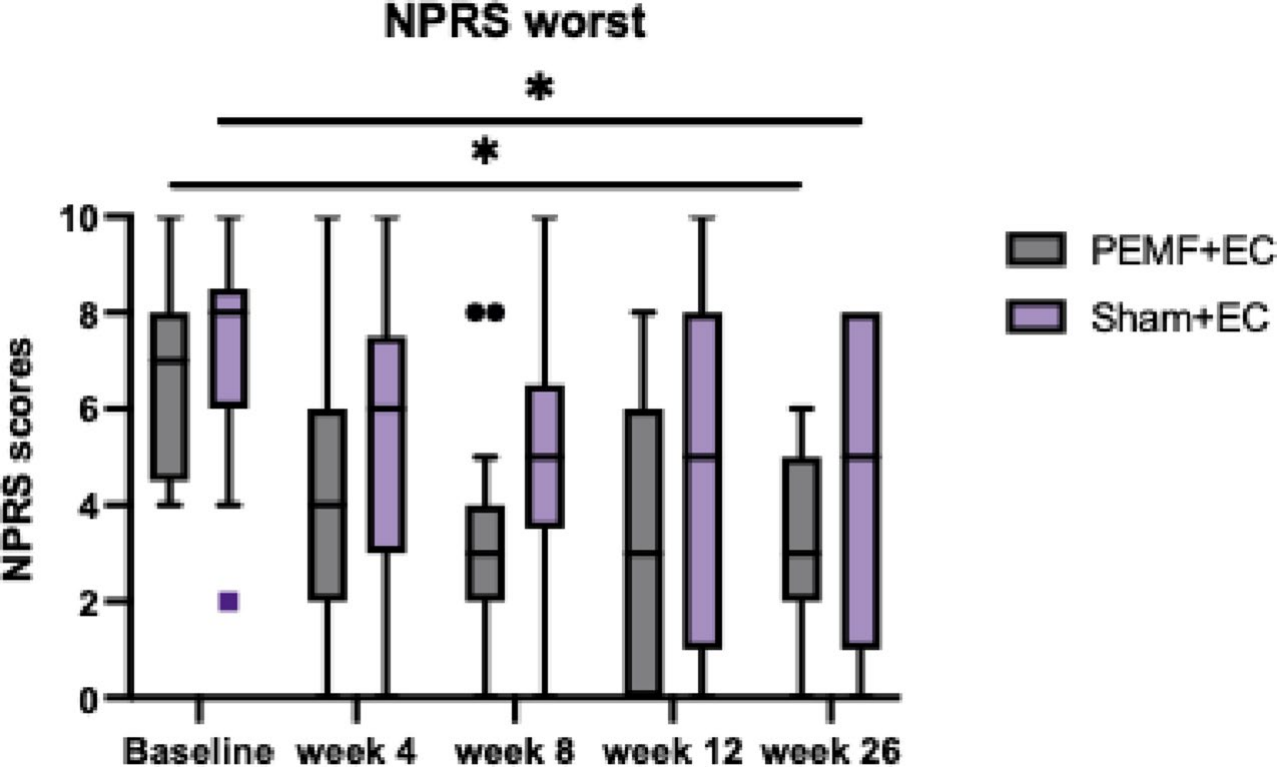
NPRS worst. Comparison of NPRS worst scores between PEMF and Sham groups across all assessment timepoints using repeated ANOVA. The data shown represent the median, interquartile range, PEMF group (*n* = 17), and Sham group (*n* = 17). NPRS worst scores significantly decreased within both treatment groups after PEMF therapy. * Represents statistically significant difference within treatment groups.

No significant differences in interaction effects were observed between treatment groups for NPRS general (F = 1.063, *P* = .368) and NPRS worst (F = 0.225, *P* = .887) (Table 3). However, both NPRS general (F = 12.161, *P* < .001) and NPRS worst (F = 15.825, *P* < .001) showed significant within-group reductions following PEMF therapy. Pre-treatment NPRS general and NPRS worst significantly decreased at week 26 in both the PEMF and Sham groups. The mean NPRS general scores for the PEMF group reduced from 3.53 to 1.53, and for the Sham group from 5.12 to 2.88 at week 26 (Fig. 5). Similarly, the mean NPRS worst scores for the PEMF group fell from 6.59 to 3.18 at week 26, while the Sham group decreased from 7.24 to 4.59 (Fig. 6).

### Short form 36 (SF36)

**Fig. 7:**
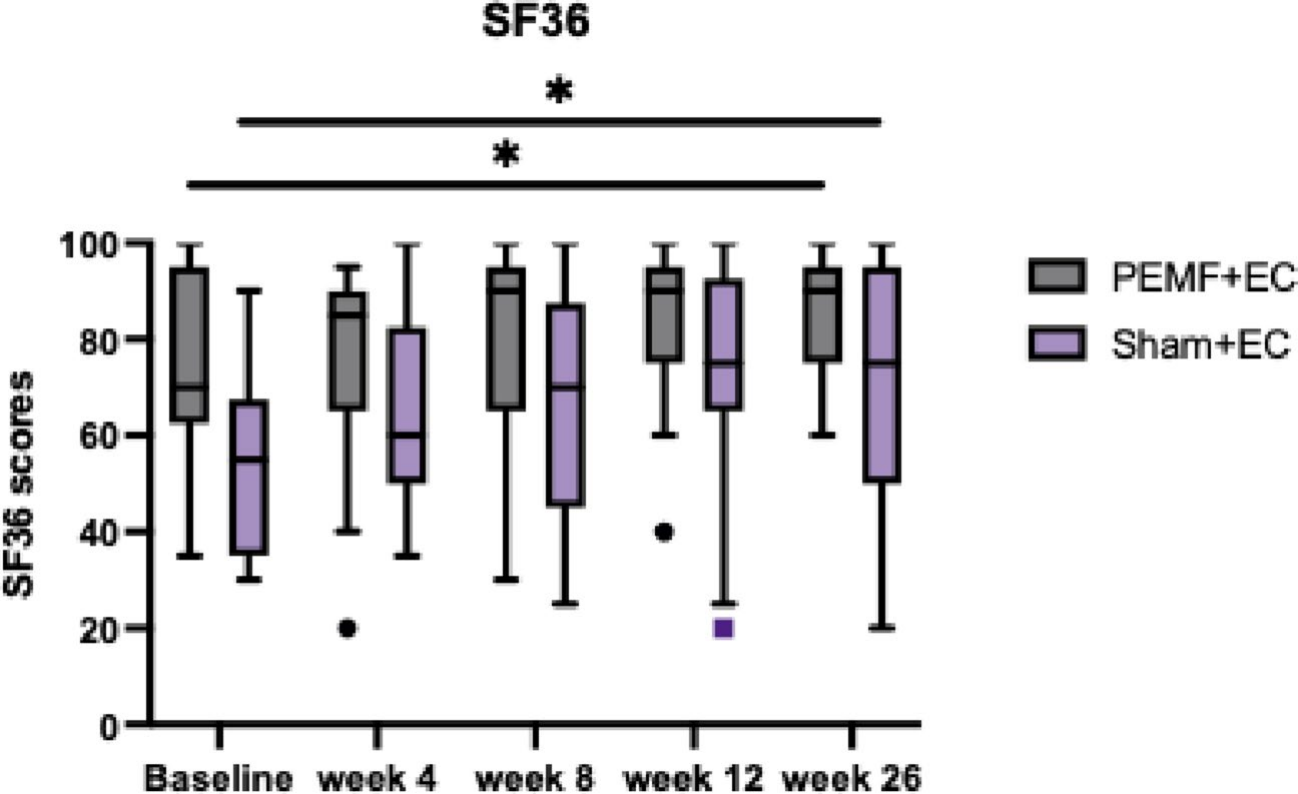
SF36. Comparison of SF36 scores between PEMF and Sham groups across all assessment timepoints using repeated ANOVA. The data shown represent the median, interquartile range, PEMF group (*n* = 17), and Sham group (*n* = 17). *SF36 scores significantly increased within both treatment groups after PEMF therapy. * Represents statistically significant difference within treatment groups.

There were no significant differences in the interaction effects on SF36 scores (F = 0.775, *P* = .498) (Table 3). However, scores within each group significantly increased over time (F = 8.215, *P* < .001). Pre-treatment SF36 improved significantly by week 26. The mean SF36 scores for the PEMF group rose from 74.12 to 85.29 at week 26, while the Sham group increased from 53.82 to 71.47 at week 26 (Fig. 7).

**Table 3:** Comparison of outcome measures between PEMF and Sham groups.

Outcome	Baseline	Follow-up				*P* (within group)
		Week 4	Week 8	Week 12	Week 26	
VISA-A						F = 9.839; *P* < .001
PEMF	59.00 ± 19.21	62.47 ± 17.28	71.24 ± 16.57	73.88 ± 18.72	79.41 ± 15.76	
Sham	53.65 ± 16.85	60.24 ± 19.24	63.12 ± 21.43	64.35 ± 24.28	64.41 ± 24.85	
P (time x groups)	F = 1.427; *P* = .243					
NPRS general						F = 12.161; *P* < .001
PEMF	3.53 ± 2.32	2.82 ± 2.01	2.06 ± 1.56	2.06 ± 2.16	1.53 ± 1.23	
Sham	5.12 ± 2.00	3.18 ± 1.88	2.94 ± 1.98	3.35 ± 2.74	2.88 ± 2.57	
P (time x groups)	F = 1.063; *P* = .368					
NPRS worst						F = 15.825; *P* < .001
PEMF	6.59 ± 2.00	4.29 ± 2.89	3.35 ± 2.21	3.29 ± 3.00	3.18 ± 2.10	
Sham	7.24 ± 2.11	5.29 ± 2.80	4.71 ± 2.76	4.47 ± 3.45	4.59 ± 3.22	
P (time x groups)	F = 0.225; *P* = .887					
SF36						F = 8.215; *P* < .001
PEMF	74.12 ± 20.63	75.29 ± 21.76	79.71 ± 19.24	83.24 ± 15.90	85.29 ± 13.17	
Sham	53.82 ± 19.17	64.12 ± 19.14	65.59 ± 25.97	71.47 ± 24.35	71.47 ± 26.85	
P (time x groups)	F = 0.775; *P* = .498					

PEMF group (*n* = 17); Sham group (*n* = 17); NPRS general: Numeric Pain Rating Scale – general on the day of review; NPRS-worst: Numeric Pain Rating Scale – worst on the day of review; SF36: Short Form 36.

## Discussion

The carry-over effects of PEMF therapy on self-reported pain, function, and quality of life were observed in VISA-A, NPRS, and SF36 scores, which were measured from baseline to week 26. According to the comprehensive statistical analysis of all assessment data points, the inclusion of active PEMF therapy alongside exercise resulted in higher VISA-A scores compared to sham PEMF combined with exercise. Although no standard treatments were administered to participants after week 12, both treatment groups demonstrated significant improvements in self-reported pain and function following the treatment period, as evidenced by VISA-A and NPRS scores.

### Pain and function

As measured by VISA-A, self-reported pain and function demonstrated significant improvement in both treatment groups following an 8-week PEMF therapy in conjunction with a 12-week home-based eccentric exercise regimen. These improvements were observed in both immediate and sustained effects. The short-term effects on pain and function have been discussed and published in a peer-reviewed scientific journal^16^. Furthermore, this article evaluated the carry-over effects by including outcome measures at 26 weeks after initiating PEMF therapy. The mean VISA-A scores improved by 20.41 points in the PEMF group, while the Sham group improved by 10.76 points at week 26. The increase in VISA-A scores for the active PEMF group surpassed the MCID threshold of 16 points^21^. Currently, practical measures such as non-steroidal anti-inflammatory drugs are employed for the temporary relief of acute pain^22^. Nevertheless, achieving long-term pain alleviation through biophysical approaches such as PEMF therapy represents a notable advancement.

### Treatment adjunct for rehabilitation

PEMF therapy can be administered in conjunction with eccentric exercise during the rehabilitation process; however, it should not serve as the sole treatment provided to patients. This is because eccentric exercise offers advantages that PEMF therapy alone is unable to deliver^23^. Eccentric exercise is essential for stimulating the synthesis of neurotrophic substances in muscles and tendons, which are relevant to the neuroregeneration of peripheral neurons^24^. Although it was unknown if eccentric exercise could modulate nerve ingrowth, it could release neuropeptides to activate the peripheral nervous system and modulate nerve plasticity, retraction, and regeneration during tendon healing. During eccentric exercise training, the force fluctuations stimulated tendon remodelling as it was more challenging to control dynamic movement with lengthening muscles^25^. The high-frequency oscillations in peak force generated a pattern of sinusoidal loading and loading of the Achilles tendon. PEMF therapy is a novel treatment option for Achilles tendinopathy to improve self-reported pain, function and quality of life, but it should not replace currently available treatment options.

### Current treatment options

Healthcare professionals might consider using PEMF therapy and eccentric exercise if traditional rehabilitation methods fail to relieve symptoms of Achilles tendinopathy. A variety of conservative treatment options are still accessible for patients diagnosed with Achilles tendinopathy. Eccentric exercise remains a fundamental component of the treatment regimen, supplemented by soft tissue mobilisation, orthoses, and modifications to activity.^10,26–28^ Chronic insertional and non-insertional Achilles tendinopathies should be evaluated after a minimum of three months of follow-up before considering surgical intervention, especially if other conservative management strategies have proven unsuccessful^29^. Ultimately, surgical intervention may be contemplated should patients endure severe symptoms that considerably hinder daily activities for a minimum of six months. The development of novel treatment adjuncts for Achilles tendinopathy, such as PEMF therapy, offers supplementary options for patients before considering surgical intervention. This approach has the capacity to optimise the utilisation of healthcare resources and diminish medical expenses associated with Achilles tendinopathy within the public healthcare system^30^.

### Limitations

The authors acknowledged certain limitations inherent in the current investigation. Firstly, participants showed a trend of improvement in pain, functionality, and quality of life throughout the study duration; however, further studies are needed to confirm the carry-over effects of PEMF therapy, especially considering the attrition caused by loss to follow-up in the long-term assessment. The absence of a statistically significant difference between treatment groups may be due to this loss to follow-up, which reduced the number of participants available for analysis. Secondly, the most effective PEMF treatment protocol for Achilles tendinopathy remains undefined. Future randomised controlled trials are recommended to evaluate the therapeutic effects of different parameters, including duration, frequency, and intensity, in patients with Achilles tendinopathy. Finally, although a biophysical mechanism underlying PEMF therapy was suggested, it could not be validated within this study’s scope. Future research could explore mechanistic studies to understand how PEMF therapy impacts Achilles tendinopathy.

## Conclusions

Pulsed Electromagnetic Field (PEMF) therapy, when combined with eccentric exercise, demonstrated clinical benefits in managing Achilles tendinopathy, evidenced by improvements across all outcomes in both treatment groups (*p* < .05). Although there were no statistically significant differences between the treatment groups for all outcomes, the group receiving active PEMF therapy exhibited clinically meaningful improvements in VISA-A scores throughout the 26 weeks. The application of PEMF therapy was well tolerated by participants, with no adverse effects reported during the trial. Further research is required to establish the most effective protocols for pulsed electromagnetic field therapy in treating Achilles tendinopathy.

## Data Availability

Raw data supporting this study’s findings are available from the corresponding author upon reasonable request.
